# Aston University's Antimicrobial Resistance (AMR) Roadshow: raising awareness and embedding knowledge of AMR in key stage 4 learners

**DOI:** 10.1016/j.infpip.2020.100060

**Published:** 2020-04-28

**Authors:** Rabia Ahmed, Amreen Bashir, James E.P. Brown, Jonathan A.G. Cox, Anthony C. Hilton, Samantha L. Jordan, Eirini Theodosiou, Tony Worthington

**Affiliations:** a School of Life and Health Sciences, Aston University, Birmingham, B4 7ET, UK; b School of Engineering and Applied Science, Aston University, Birmingham B4 7ET, UK

**Keywords:** Antimicrobial resistance, Health education, Public engagement

## Abstract

Antimicrobial resistance (AMR) is a global healthcare problem and therefore raising awareness within young learners is imperative. An AMR roadshow was designed to take key stage 4 students' learning ‘out of the classroom’, assess pre-existing knowledge of AMR and determine the impact of the roadshow on knowledge retention. Knowledge and subsequent retention were measured pre- and post-event through a standardised questionnaire. The roadshow significantly improved knowledge and understanding of AMR, which was retained for a minimum of twelve weeks. Engaging and interactive strategies addressing key health issues provide a positive learning experience which contributes to retained knowledge in young learners.

## Introduction

Antimicrobial resistance (AMR) is a significant world-wide public health problem. [1] It is crucial therefore to raise public awareness of this growing global healthcare issue. The general public, including young learners, has been shown to have misconceptions regarding antibiotics and their misuse. [2,3] Raising awareness of AMR therefore, not only within the healthcare system but also within society, plays an important role in effective antimicrobial stewardship. [3] Antibiotic awareness campaigns have been shown to raise public awareness of AMR through communication using social media, fact sheets, posters and videos. [4].

Theatre performance is an alternative educational campaign for increasing public awareness of health issues such as HIV/AIDS [5] and smoking. [6] In our recent study, [2] it was demonstrated that Educational Theatre in the form of a live three-act play significantly impacted upon general public knowledge, understanding and attitudes towards AMR. A key finding of this study was that the majority of students attending the live performance at the Think Tank, Birmingham, UK were of the key stage 4 (KS4) age group (ages 14–16) and their knowledge and understanding towards AMR were significantly improved post-event. However, it was beyond the scope of the study to ascertain whether the positive shift in knowledge was retained over time. In addition to engaging initiatives, such as theatre performance, it is also accepted that primary and secondary school student knowledge is positively influenced by ‘learning outside of the classroom’ (LOtC). [7] Such opportunities, which include day visits to organisations, investigations conducted in the local area, including education departments, and drama productions are valued by both learners and teachers. [7].

The aim of this current investigation was to add to our previous Educational Theatre study [2] and develop an interactive collection of workshops at Aston University (Birmingham, UK), which offered LOtC to KS4 students with the aim of raising awareness and understanding of AMR. The AMR roadshow comprised interactive workshops in the disciplines of Microbiology, Pharmacy and Engineering; all of which fed into the overall theme of AMR. Student knowledge relating to AMR was determined prior to the event and directly post-event using a standard AMR questionnaire. To add to our previous study, [2] students' retention of knowledge and understanding was determined 6 and 12 weeks post-event [8,9] using the same questionnaire.

## Methods

### Participants in the AMR roadshow

Three academies participated in the AMR roadshow: Wood Green Academy (Wood Green Rd, Wednesbury WS10 9QU), Walsall Academy (Lichfield Rd, Bloxwich, Walsall WS3 3LX) and Aston University Engineering Academy (AUEA), (1 Lister St, Birmingham B7 4AG).

### The AMR roadshow

The AMR roadshow was a one-day event delivered at Aston University, Birmingham, UK, in the fields of Microbiology, Pharmacy and Biochemical Engineering. The event comprised 4 interactive workshops: (1) Introduction to Microbiology and AMR; (2) Infection and the Superbug Methicillin Resistant *Staphylococcus aureus* (MRSA); (3) Antibiotics; (4) Molecules to Man. KS4 students from the Birmingham region were invited to the AMR roadshow. Participants' pre-existing knowledge of antibiotics and AMR was assessed through a standardised questionnaire. Each questionnaire was assigned with a unique code identifier. The pre-event questionnaire was issued to all participants on attendance at the AMR Roadshow for completion in workshop (1). Post-event questionnaires were issued for completion and collection following the AMR Roadshow at the end of the event. To determine retention of AMR knowledge over an extended period post-event, identical questionnaires containing the unique code identifiers were issued to academies for subsequent distribution to their students at 6 and 12 weeks. Students within the academies completed the questionnaires independently during a designated tutorial period.

### The AMR workshops

(1) Determining pre-existing knowledge of Microbiology and AMR.

In this introductory session, students completed the pre-event questionnaire which collated demographic information (age, gender, postcode, ethnic group) and existing knowledge and understanding of antibiotics and AMR. The questionnaire comprised sixteen questions; eleven multiple choice (4 options with single correct answer) which were corrected to binary (correct/incorrect) for analysis, and 5 using standard 5-point Likert scales where 1="very unlikely/not important” and 5="very likely/very important”. The content of the questionnaire related to aspects of antibiotics and AMR that the student may (a) already know (b) be acquired through the subsequent interactive workshops.

(2) Infection and the Superbug Methicillin Resistant *Staphylococcus aureus* (MRSA).

An interactive session on MRSA was delivered which included: the epidemiological aspects; acquisition of resistance; the chain of infection and breaking the chain of infection (PPE, hand washing and hand hygiene, cleaning and disinfection, patient placement, MRSA screening). For the practical interactive component, students practiced the World Health Organisation hand washing technique to demonstrate effective handwashing using ‘Glitter Bug Cream’ (UV disclosing lotion) for hand contamination and UV boxes to highlight results post-handwashing. This aspect of the workshop was an engaging activity and valuable exercise used to demonstrate effective hand washing technique; a key element in infection control procedures and ‘breaking the chain of infection'.

(3) Antibiotics

An interactive session was delivered comprising the history of antibiotics, how antibiotics work, AMR on a global scale, the 5 mechanisms through which bacteria become resistant to antibiotics and measures that can be undertaken to help prevent AMR. For the practical aspect, students examined antibiotic disk diffusion assays and measured zones of inhibition (ZOI) of Ampicillin (10μg), Gentamicin (10 μg), Ciprofloxacin (1 μg) and Trimethoprim (2.5 μg) against *Staphylococcus aureus* ATCC 6538. To raise awareness of the potential antimicrobial efficacy of naturally occurring substances, students examined agar diffusion plates inoculated with *S. aureus* ATCC 6538 and measured the ZOI of: Manuka honey, garlic, ginger and lemon and compared them to the ZOI achieved for the antibiotics tested.

(4) Molecules to Man

This workshop focused on new drug discovery (Pharmacy and Biochemical Engineering), pre-clinical and clinical development of antimicrobials, and manufacturing issues. This was illustrated with the discovery of penicillin and the processes involved in producing large quantities of the drug. The interactive component comprised two main activities: the production challenges following drug discovery (i.e. creation of robust processes within short time limits) through the construction, within 30 minutes, of an accurate 3D molecular model of penicillin, and secondly, the role of pharmacokinetics in drug design. Students were asked to make coloured beads using alginate, a component employed in drug formulation. The disposition of a pharmaceutical compound within the body (i.e. absorption, distribution, metabolism and excretion (ADME)) was then demonstrated using standard chemistry lab ware.

### Statistical analysis of the data

The non-parametric Wilcoxon matched-pairs test was used to compare the Likert scores for pre- and post-performance responses for each question (GraphPad Prism version 7.00 for Windows, GraphPad Software, La Jolla California USA).

## Results

### Participants and demographics

The AMR roadshow was attended by 159 KS4 students: Wood Green Academy (n=30), Walsall Academy (n=52), AUEA (n=77). The gender split was male (n=110) and female (n =49). Participants were aged 15 (n=60) or 16 (n=99). Participant ethnicity included: UK and Ireland (n= 62): Asia (n=64); Africa (n=24) Caribbean (n=5); Middle East (n=4). Table 1 presents the questions (MCQ and Likert Scale) to assess KS4 participant knowledge pre and post-AMR roadshow and retained knowledge 6 and 12-week post event. Only data from participants completing all questionnaires were used in the final analysis. The total number of fully completed questionnaires were: pre-event (n=80), post event (n=74), six weeks (n=70), 3 months (n=72).

**Table 1:** MCQ and Likert Scale questions answered by participants

	Question	Type of question
1	A bacterium can grow and divide every? …. .(20 minutes)	MCQ
2	Penicillin was first purified by? …. .(Howard Florey and his team)	MCQ
3	How likely are you to use all six stages of handwashing	Likert
4	The first commercial plant for the large scale production of penicillin was designed by a team of …. (Chemical Engineers)	MCQ
5	How likely are you to wash your hands properly after a potential exposure to a bug (e.g. blowing your nose, going to the toilet)?	Likert
6	How important do you think it is to wash your hands properly throughout the day?	Likert
7	On average how long does it take to get a drug from bench to market …. (10–15 years)	MCQ
8	How likely are you to use a natural product when you have bacterial infection instead of taking antibiotics?	Likert
9	What is MRSA?...(an antibiotic resistant bacterium)	MCQ
10	What is the single most effective way of preventing the spread of MRSA in hospitals?...(wash your hands)	MCQ
11	How important do you think proper hand washing is in preventing the spread of antibiotic resistance?	Likert
12	Antibiotic resistance is when? …. (bacteria become resistant to antibiotics)	MCQ
13	How many stages are there to proper handwashing?...(6)	MCQ
14	Antibiotic resistance is caused by? …. (people)	MCQ
15	Which of the following natural products does not exhibit antimicrobial activity?...(lettuce)	MCQ
16	What is the minimum time you should wash your hands for (e.g. after going to the toilet)?...(20 seconds)	MCQ

**Figure 1:**
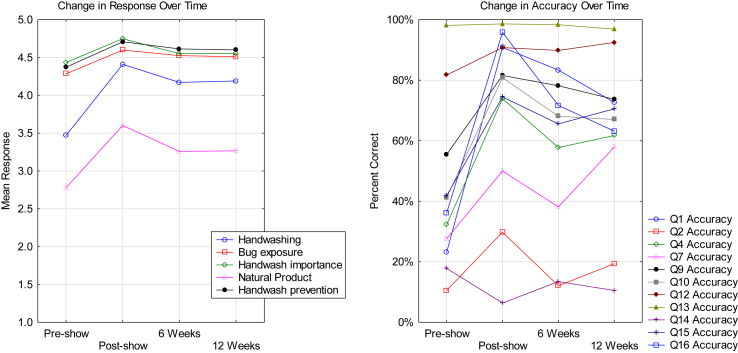
Student questionnaire responses. **Left**: Change in response over time to Likert Scale questions (Q3, 5, 6, 8, 11 (Table 1). **Right**: Percentage of correct MCQ responses and change in accuracy over time.

Student questionnaire responses relating to knowledge and understanding of AMR through Likert Scale and MCQ questions over time are shown in Figure 1.

Regarding the MCQ responses, prior to the roadshow, 3 out of 11 (27%) were answered correctly by 50% or more of the KS4 cohort, whilst directly after the AMR roadshow 9 out of 11 (82%) were answered correctly by 50% or more of the students. Directly post-AMR event, a positive increase in knowledge was demonstrated in 91% of the MCQ with only Q14 (see table 1) demonstrating a decrease in knowledge.

Pre-AMR roadshow MCQ questionnaire responses demonstrated that participants had an existing knowledge (as indicated by >50% initial correct responses) of MRSA, stages of handwashing and what antibiotic resistance means. Initial knowledge regarding issues including: minimum time for effective handwashing, natural antimicrobials, antibiotic production and preventing the spread of antimicrobial resistant microorganisms was less embedded, with correct responses ranging from 10% to 42%. Q2 (Penicillin was purified by …. ?) was answered incorrectly by 90% of the participants prior to the AMR roadshow alongside Q14 (antibiotic resistance is caused by …. ?) which was answered incorrectly by 81% of the students.

Regarding the Likert Scale questions, a good level (mean response >4) of pre-existing knowledge relating to the importance of handwashing in preventing the spread of AMR (Q11), washing your hands properly (Q6) and following potential exposure to microorganisms (Q5) was demonstrated. Less knowledge (mean response <3.5) was demonstrated relating to the 6-stages of handwashing (Q3) and the potential use of natural products to treat infection.

There was no significant difference in responses based on academy or gender (p> 0.05). Furthermore, ethnicity was not significantly associated with the participants' questionnaire responses; participants from Europe and mixed ethnic backgrounds demonstrated an increased knowledge of AMR across the study period, whilst those from Asia and the Caribbean displayed a similar trend but with an increased retained knowledge at twelve weeks. The AMR roadshow improved knowledge and understanding of AMR (as demonstrated by a positive change in accuracy of responses to the 11 MCQ questions and a positive change in response to the 5 Likert Scale questions) across the KS4 student cohort. Increased accuracy to 91% MCQ questionnaire responses and 100% positive change in response to Likert Scale questions was demonstrated at 12 weeks post event.

## Discussion

The AMR roadshow provided an interactive LOtC platform to determine existing knowledge and understanding of AMR amongst KS4 students and also the impact of the roadshow in knowledge enhancement and retention over a 12-week period. It has been shown in previous studies that adopting a variety of different ‘teaching’ platforms to engage the public and young learners is central to raising awareness of important healthcare issues. [2,[4], [5], [6]].

In our previous report, [2] it was demonstrated that knowledge of AMR amongst KS4 students who attended our AMR Educational Theatre event at the Think Tank, Birmingham, was clearly influenced by the event, however sustained retention of knowledge was not determined. This current study has demonstrated that, through participation in the AMR roadshow and assessment of knowledge through a standardised questionnaire, under controlled conditions and over an extended period of time, knowledge of AMR is improved and embedded for a minimum of 12 weeks in young learners.

The findings from this study demonstrated that the level of pre-existing knowledge and awareness amongst the KS4 academy cohort with regards to antibiotics and AMR varied considerably. This was not entirely surprising considering the differences in curriculum between academies, triple science or combined science studies students, and natural variation in learning ability amongst the cohort. Indeed, other factors relating to learning ability, which were not investigated in this current study, may include aspects such as attention disorder (diagnosed or undiagnosed), support at home from parents and family and general mental health and wellbeing. All of these factors are likely to contribute to some extent to the broad range of pre-existing AMR knowledge and overall learning. Furthermore, this current study included only KS4 students from academies; the study may have benefitted from inclusion of KS4 students from non-academy institutions to compare learning, knowledge enhancement and retention. Nonetheless, through the use of inspirational educational platforms, such as the Aston AMR roadshow, whereby students undertake LOtC and engage through active learning with a hands-on interactive approach, this study has demonstrated that knowledge is clearly enhanced and retained over a 12 week period. Our findings are in line with those of Freeman *et al.* (2013) [10] who demonstrated that active learning significantly increases student performance in STEM subjects. A 6 and 12 week post-AMR roadshow period to assess knowledge retention was chosen in this current study as this time frame has been adopted in previous studies. [8,9] In the study by Young and King (2000), a modest increase in theoretical knowledge (but a decrease in practical skill performance) relating to advanced life support skills in Nursing was demonstrated over a 12 week period. Furthermore, Smidt *et al.* [9] investigated retention of knowledge in teachers taught to deliver sign language to children with disabilities during a one-day training workshop. This was assessed at 6 and 12 weeks post sign language workshop event. They demonstrated that participants were able to learn language signs during the one-day workshop, however sign knowledge decreased after 6 and 12 weeks. In our study, we found the contrary in that knowledge was significantly improved and retained for up to 12 weeks after our one-day AMR workshop. However, numbers, participants and demographics and subject material differ from study to study.

What is clear from our findings is that young learners are aware of basic AMR issues, including MRSA and the importance of handwashing, but are less aware of ‘how’ to wash hands properly or ‘how’ AMR occurs. Many students thought that ‘people’ become resistant to antibiotics, which is a common misconception and were not familiar with the potential use of natural products, e.g. Manuka Honey in treatment of infection. Currently, there is little known about the most effective mechanism to raise awareness of important healthcare issues, including AMR, within the public, but the body of research and evidence is increasing and includes platforms such as performing arts, educational theatre, theatre production and LOtC. [2,[4], [5], [6], [7]] Different individuals learn in different ways, but what is also clear from our recent study [2] and the data generated from the AMR roadshow, is that these engaging and inspiring learning strategies make a positive impact, resulting in increased knowledge which is embedded and retained over time.

## Ethics approval and consent to participate

An application was submitted to the University Research & Ethics Committee and considered by the Life & Health Sciences (LHS) Ethics Committee under application #1107. The committee granted that the study, as an evaluation of an educational resource, was eligible for self-certification. Consent from parents of KS4 participants was sought prior to the event.

## Consent for publication

Not applicable.

## Availability of data and material

The datasets used and analysed during the current study are available from the corresponding author on reasonable request. The AMR roadshow information and workshop booklet is available upon request. On-line video links which provide underpinning and additional information to support the AMR roadshow can be found at:

https://www.youtube.com/watch?v=qRIR-X7N2VI

https://www.youtube.com/watch?v=eQ0Z1gzhndg

https://www.youtube.com/watch?v=00RvoJSBn1A

https://www.youtube.com/watch?v=zeRmpaCeAmU

## Conflicts of interest

The authors declare they have no competing interests.

## Funding

The work was supported by the UK Engineering and Physical Sciences Research Council: Bridging the Gaps between Engineering and Physical Sciences in Antimicrobial Resistance, as part of the UK cross-Research Council Initiative on Antimicrobial Resistance (AMR) Grant number EP/M02735X/1.

## Author's contributions

RA, AB, JC, AH, SJ, ET and TW authored the AMR roadshow and contributed to the event expert panel. JB contributed to the development and analysis of the event evaluation materials. All authors contributed to, read and approved the final manuscript.
